# Individual variations and sex differences in hemodynamics and percutaneous arterial oxygen saturation (SpO_2_) in Tibetan highlanders of Tsarang in the Mustang district of Nepal

**DOI:** 10.1186/s40101-022-00282-4

**Published:** 2022-03-15

**Authors:** Takayuki Nishimura, Hiroaki Arima, Sweta Koirala, Hiromu Ito, Taro Yamamoto

**Affiliations:** 1 Department of Human Science, Kyushu University, 4-9-1 Shiobaru, Minami-ku, Fukuoka, 815-8540 Japan; 2 Department of International Health and Medical Anthropology, Institute of Tropical Medicine, Nagasaki University, 1-12-4 Sakamoto, Nagasaki, 852-8523 Japan; 3 Nepal Development Society, Pokhara Metropolitan City, Ward 29, Naubise, Kaski District, Kathmandu, Nepal

**Keywords:** High-altitude adaptation, SpO_2_, Individual variation, Sex difference, Tibetan highlanders

## Abstract

**Background:**

Many studies have indicated specific low-hemoglobin (Hb) adaptation to high altitude in the Tibetan population, but studies focusing on physiological variations within this population are limited. This study aimed to investigate the relationships between SpO_2_ and related factors, including individual variations and sex differences, to assess the generality of high-altitude adaptation in the Tibetan population of Tsarang.

**Methods:**

The participants were 31 male and 41 female community-dwelling people aged ≥18 years living in Tsarang, in the Mustang district of Nepal. Height, weight, SpO_2_, Hb concentration, finger temperature, heart rate, and blood pressure were measured. Lifestyle information was obtained by interview.

**Results:**

Men had significantly higher systolic blood pressure (*p* = 0.002) and Hb (*p* < 0.001) than women. There was no significant correlation between SpO_2_ and other parameters in men. In women, SpO_2_ was negatively correlated with heart rate (*p* = 0.036), Hb (*p* = 0.004), and finger temperature (*p* = 0.037). In multiple regression analysis, a higher SpO_2_ was marginally correlated with lower age (*β* = −0.109, *p* = 0.086) and higher Hb (*β* = 0.547, *p* = 0.053) in men. In women, higher SpO_2_ was significantly correlated with lower heart rate (*β* = −0.045, *p* = 0.036) and Hb (*β* = −0.341, *p* = 0.018). Mean hemoglobin (95% confidence interval) was 13.6 g/dl (13.1–14.0 g/dl), which is lower than that found previously in Andeans and almost equal to that in Japanese lowlanders measured using the same device. In some participants of both sexes, hemoglobin was >17.0 g/dl.

**Conclusion:**

Higher SpO_2_ was marginally correlated with younger age and higher Hb in men and with lower heart rate and lower Hb in women. Hemoglobin concentration was similar to that found previously in lowlanders, but higher in some individuals. These results indicate individual variation and sex differences in the hemodynamics of high-altitude adaptation in Tibetan highlanders of Tsarang, as well as low-Hb adaptation to high altitude equal to that of other Tibetans.

## Background

High-altitude adaptation has been researched and discussed for over 100 years. Previous studies have revealed specific populational differences in high-altitude adaptations among such as Andean, Tibetan, and Ethiopian populations [1–5]. It is well known that Tibetan highlanders have lower hemoglobin (Hb) and SpO_2_ levels than Andean highlanders [2, 4, 5]. Recent genomic analysis has clarified the mechanism of low-Hb adaptation to high altitude in Tibetans [1, 6–8], who were termed ‘King of the Mountains’ by Edward et al. [9] because of their ability to live and reproduce successfully at high altitude.

Although populational differences have been well discussed, there is limited knowledge of physiological variations in highlanders [3, 10]. From the perspective of physiological anthropology, various studies have conducted field research to investigate variation in physiological response from the perspective of acute hypobaric hypoxia in lowlanders [11, 12] or of physiological variation in highlanders [6, 13, 14]. In our previous field research, we investigated the health status of Tibetans in Tsarang village (altitude, 3570 m) [6]. The Tsarang village is highly preserved due to its location in Mustang district, neighboring the Tibetan area of China. Physiological data from this region are limited because the Mustang district was isolated from other parts of Nepal until 1992 [15–17]. We have also investigated individual variations and sex differences in the percutaneous arterial oxygen saturation (SpO_2_) of young Andean highlanders in Bolivia (altitude, 3700–4000 m). As these studies share common measurement devices and protocols [6, 14], the physiological states can be compared between these two populations.

Therefore, the aim of the study was to investigate individual variation and sex differences in SpO_2_ and related factors, to assess the generality of high-altitude adaptation in the Tibetan population of Tsarang.

## Methods

This cross-sectional study was conducted in Tsarang village (altitude, 3570 m), Dhaulagiri zone, in the Mustang district of Western Nepal in July 2017. We recruited 188 participants (85 men and 103 women, age ≥ 18 years). To enable comparison with our previous study of highlanders in Bolivia, we selected those aged 18–40 years from this group and analyzed the data of a final total of 31 men and 41 women.

After explaining the experiment and obtaining informed consent, each participant’s height, weight, SpO_2_, Hb concentration, finger temperature, heart rate, and blood pressure were measured. All parameters were measured at room temperature (22–24 °C) with the participants wearing traditional clothes.

After anthropometric measurements for height and weight, SpO_2_ was measured by a finger pulse oximeter (Masimo Radical V 5.0; Masimo Corp, Irvine, CA). Hb concentration and finger temperature were measured on the inner surface of the index finger using an ASTRIM FIT health monitoring analyzer (Sysmex; Kobe, Japan). Systolic blood pressure (SBP), diastolic blood pressure (DBP), and heart rate were measured in the left arm in the resting condition by a digital automatic blood pressure monitor (HEM-7210, OMRON; Kyoto, Japan).

Height (cm) and weight (kg) were measured with clothing and without shoes, and body mass index (BMI) was calculated as weight/height squared (kg/m^2^). Information about physical activity (walking or doing any equivalent amount of exercise activity more than 30 min per day: yes/no), current smoking (having one or more cigarettes per day: yes/no), and alcohol use (some alcohol consumption one or more days per week: yes/no) was obtained by interview. Details of the research and data collection methods have been described elsewhere [6, 15].

### Statistical analysis

Variables are presented as the mean and 95% confidence interval (95% CI). Student’s *t*-test and Fisher’s exact test were used for comparisons between men and women. Pearson’s correlation analysis was used to assess correlations between SpO_2_ and other parameters for men, for women, and for total participants. Multiple regression analysis was used to assess correlations between SpO_2_ and related parameters (sex, age, heart rate, DBP, Hb, and finger temperature) for men, for women, and for total participants. These parameters were added to the above model because their *p* values were <0.15 in a simple correlation analysis between SpO_2_ and other parameters in each sex. Finger temperature data were missing in 1 man and 1 woman, and Pearson’s correlation analysis and multiple regression analysis omitted data of 1 man and 1 woman. All analyses were performed using the Statistical Analysis System software package version 9.4 (SAS Institute, Cary, NC).

## Results

Table 1 summarizes the characteristics of the 72 participants. Height and weight were significantly higher in men than women (*p* < 0.001), but there was no significant difference in BMI. SBP and Hb were also significantly higher in men than women (*p* = 0.002 and *p* < 0.001, respectively). There was no significant difference between men and women in terms of SpO_2_, heart rate, DBP, or finger temperature. Regarding lifestyle factors, men had significantly higher rates of smoking and alcohol use (*p* = 0.043 and *p* < 0.001, respectively).

**Table 1:** Characteristics of the study population

	Total (*n* = 72)	Men (*n* = 31)	Women (*n* = 41)	*P*-value
	Mean (95% *CI*)			
Age (y)	31.2 (29.7–32.7)	32.5 (30.5–34.6)	30.1 (28.1–32.3)	0.113
Height (cm)	158.4 (156.2–160.5)	165.2 (162.7–167.8)	153.2 (151.0–155.4)	< 0.001
Weight (kg)	56.7 (54.4–59.1)	62.2 (59.4–65.0)	52.6 (49.5–55.6)	< 0.001
BMI (kg/m^2^)	22.6 (21.8–23.4)	22.9 (21.7–24.0)	22.4 (21.2–23.5)	0.544
Heart rate (bpm)	81.0 (78.4–83.6)	79.2 (75.5–82.9)	82.3 (78.6–86.1)	0.241
SBP (mmHg)	117.6 (114.6–120.6)	123.0 (118.4–127.6)	113.6 (109.9–117.2)	0.002
DBP (mmHg)	75.6 (73.1–78.0)	77.7 (74.0–81.3)	74.0 (70.6–77.3)	0.138
SpO_2_ (%)	92.4 (92.0–92.8)	92.5 (91.8–93.2)	92.4 (91.9–92.9)	0.820
Hemoglobin (g/dl)	13.6 (13.1–14.0)	14.6 (14.1–15.1)	12.7 (12.2–13.4)	< 0.001
Finger temperature (°C)	33.8 (33.1–34.6)	34.4 (33.4–35.5)	33.4 (32.3–34.5)	0.179
	% (95% *CI*)			
Current smoking (yes)	22.5 (13.5–34.0)	35.5 (18.6–52.3)	12.5 (2.3–22.8)	0.043
Alcohol drinking (yes)	16.9 (8.2–25.6)	35.5 (18.6–52.3)	2.5 (0.0–7.3)	< 0.001
Physical activity (yes)	74.6 (64.5–84.8)	67.7 (51.3–84.2)	80.0 (67.6–91.0)	0.279

*95% CI* 95% confidence interval, *BMI* Body mass index, *SBP* Systolic blood pressure, *DBP* Diastolic blood pressure, *SpO*_*2*_ Saturation of percutaneous oxygen

**Fig. 1:**
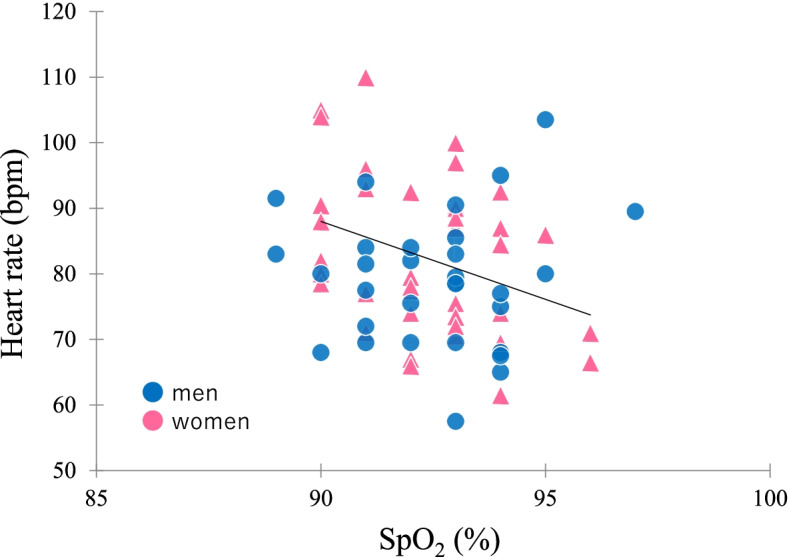
Scatter plot of SpO_2_ and heart rate in Tibetan highlanders living in Tsarang. SpO_2_ was negatively correlated with heart rate (*r* = −0.329, *P* = 0.036) in women. The solid line indicates the trend for women

In men, there was no significant correlation between SpO_2_ and other parameters (Fig. 1, Table 2). In women, SpO_2_ was negatively correlated with heart rate, Hb, and finger temperature (Fig. 1, Table 2).

**Table 2:** Simple correlation coefficients between SpO_2_ and other parameters

	Total (*n* = 72)		Men (*n* = 31)		Women (*n* = 41)	
	*r*	*p-*value	*r*	*p-*value	*r*	*p-*value
Age (y)	−0.259	0.028	−0.247	0.180	−0.289	0.067
BMI (kg/m^2^)	−0.047	0.698	−0.197	0.288	0.060	0.710
Heart rate (bpm)	−0.171	0.150	0.054	0.773	−0.329	0.036
SBP (mmHg)	−0.134	0.263	−0.075	0.689	−0.226	0.155
DBP (mmHg)	−0.100	0.403	0.086	0.645	−0.259	0.102
Hemoglobin (g/dl)	−0.128	0.283	0.252	0.172	−0.433	0.004
Finger temperature (°C)^a^	−0.156	0.196	0.082	0.667	−0.332	0.037

*SpO*_*2*_ Saturation of percutaneous oxygen, *BMI* Body mass index, *SBP* Systolic blood pressure, *DBP* Diastolic blood pressure

^a^Men (*n* = 30), women (*n* = 40)

In multiple regression analysis, higher SpO_2_ was marginally correlated with lower age and higher Hb in men (Table 3). In women, higher SpO_2_ was significantly correlated with lower heart rate and Hb (Table 3). Higher SpO_2_ was marginally related to higher age in all participants and in men (Table 3).

**Table 3:** Multiple regression analysis between SpO_2_ and other parameters

	Total (*n* = 70)			Men (*n* = 30)			Women (*n* = 40)		
	*β* (95% *CI*)	*p-*value	*r*^2^	*β* (95% *CI*)	*p-*value	*r*^2^	*β* (95% *CI*)	*p-*value	*r*^2^
Sex (women/men)	−0.385 (−1.356, 0.585)	0.431		–	–		–	–	
Age (y)	−0.060 (−0.131, 0.011)	0.094		−0.109 (−0.235, 0.017)	0.086		−0.016 (−0.094, 0.062)	0.676	
Heart rate (bpm)	−0.027 (−0.070, 0.011)	0.204		−0.012 (−0.120, 0.095)	0.813		−0.045 (−0.087, −0.003)	0.036	
DBP (mmHg)	−0.003 (−0.048, 0.041)	0.885		0.048 (−0.055, 0.151)	0.349		−0.010 (−0.058, 0.038)	0.672	
Hemoglobin (g/dl)	−0.099 (−0.360, 0.162)	0.450		0.547 (−0.007, 1.102)	0.053		−0.341 (−0.619, −0.063)	0.018	
Finger temperature (°C)	−0.051 (−0.190, 0.089)	0.472		0.102 (−0.161, 0.365)	0.433		−0.089 (−0.241, 0.064)	0.247	
			0.120			0.223			0.365

*β* Standardized regression coefficient, *95% CI* 95% confidence interval, *DBP* Diastolic blood pressure, *r*^2^ Coefficient of determination for model

## Discussion

Previous studies have indicated low-Hb adaptation to high altitude in Tibetans [1–5, 10, 18–22]. In the present study, we present physiological data of hemodynamic parameters (focusing primarily on SpO_2_) and individual variation and sex differences in isolated Tibetan highlanders living in Tsarang village.

The present study found no significant difference in SpO_2_ between men and women (Table 1). Beall et al. [23] reported lower SpO_2_ in male than female Tibetans living at an altitude of 3800–4200 m (mean SpO_2_: 90.2% at 3800 m, 89.2% at 3850 m, 88.7% at 4065 m, 88.9% at 4200 m) and sex differences in SpO_2_ tended to be greater at higher altitudes. SpO_2_ was relatively higher (92.4% at 3570 m) in the present study, and a similar study reported no sex differences in Tibetans at 3658 m [24]. Accordingly, sex differences in SpO_2_ might not be apparent in the Tibetan population at altitudes below 3700 m.

Higher Hb and blood pressure in men compared with women have been reported in a sea-level environment [25–27]. Similarly, Hb and SBP were both significantly higher in men than in women in the present study (Table 1), consistent with the result for Hb reported in a previous study of Tibetan and Andean highlanders [3]. These results suggest that the same sex differences in Hb and SBP are present in both highlanders and lowlanders.

The present results found no significant correlation between SpO_2_ and heart rate in men, but that SpO_2_ negatively correlated to heart rate in women (Fig. 1, Table 2). In women, multiple regression analysis showed that lower SpO_2_ was significantly correlated with higher heart rate after adjusting for covariates (Table 3). Heart rate decreases after long-term high-altitude exposure [28]; however, the present results indicate that lower SpO_2_ potentially evoked a higher heart rate for greater oxygen delivery even in Tibetan highlanders, and especially in women. This sex difference may indicate that in the case of higher Hb in men than women and higher blood flow for oxygen delivery in Tibetans [29, 30], lower SpO_2_ is not necessary to evoke a higher heart rate in men. Thus, although the association between SpO_2_ and heart rate is similar between Tibetan men and women, further studies that add men’s data and blood flow measurements are required to assess this complex association.

In men, multiple regression analysis revealed that lower SpO_2_ was marginally correlated with lower Hb after adjusting for covariates (Table 3). In contrast, in women, lower SpO_2_ was significantly correlated with higher Hb after adjusting for covariates (Table 3). This negative correlation in women is reasonable because higher Hb enables higher oxygen delivery, even if SpO_2_ is lower. However, as there was a weak positive correlation between SpO_2_ and Hb in men, the inverse result is difficult to explain. As men had higher Hb than women, this positive correlation might indicate better hemodynamic adaptation to high altitude or increasing pulmonary arterial pressure in men. Contrary to the present results, Beall et al. [3] reported a negative correlation between SpO_2_ and Hb in Tibetan men but not in Tibetan women at 3800–4065 m. This inconsistency in sex differences might be due to inconsistency in the studied high-altitude environments and population differences between TAR (Tibet autonomous region) and Tibetans living in Tsarang. In addition, Beall et al. [23] also suggested the effect of age and sex interactions on SpO_2_ in Tibetans in TAR, and they also recently reported aging changes of SpO_2_ in Tibetan women in Tsarang [31]. Similarly, our results also showed an aging effect of SpO_2_, in men and overall (Table 3). Taken together, the present results and those of previous studies suggest sex differences in hemodynamics and SpO_2_ in Tibetan highlanders of Tsarang. Further studies are necessary to clarify the biological mechanism of this complex association in greater detail.

We have previously reported sex and individual differences in hemodynamics and SpO_2_ in young Andean highlanders in Bolivia (altitude, 3700–4000 m) in a study of a similar duration, using the same protocol [14]. SpO_2_ was higher in the present study (mean [95 % *CI*], 92.4% [92.0–92.8%]) compared with the Andean highlanders (91% [90.0–91.0%]). Although previous studies have reported lower SpO_2_ in Tibetans than in Andeans at similar altitudes [1, 3], Tsarang is located at 3570 m and the environment is moderately hypoxic compared with Bolivia (3700–4000 m), which might be the reason why Tibetan in Tsarang could maintain higher SpO_2_.

**Fig. 2:**
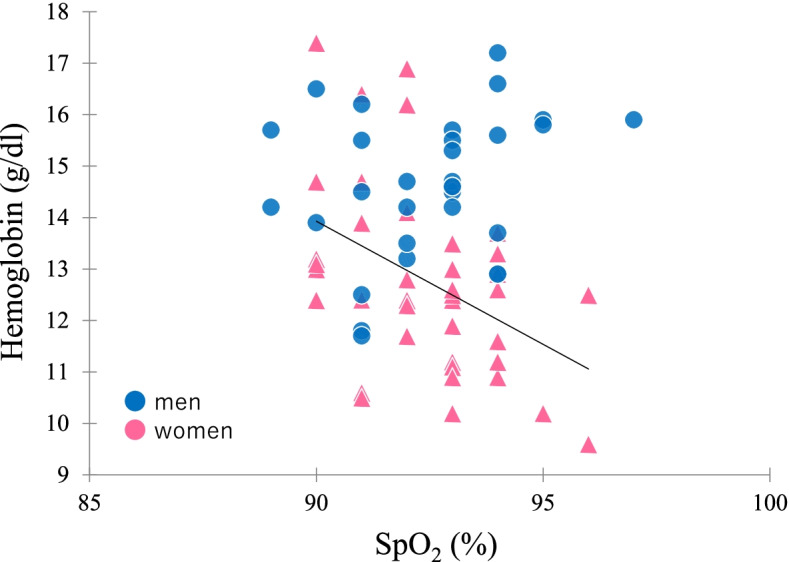
Scatter plot of SpO_2_ and hemoglobin in Tibetan highlanders living in Tsarang

Typically, among lowlanders, men have higher skin temperature and peripheral blood flow than women, in a thermoneutral environment [32, 33]. Another study found higher finger temperature in Andean men than in Andean women [14]. Interestingly, there was no significant sex difference in finger temperature in the present study, and it was slightly higher in Tibetans than in Andeans in our previous studies [14]. Previous studies have reported that Tibetan highlanders had high nitric oxide (NO) concentration and blood flow for oxygen delivery [29, 30], which suggests that Tibetan highlanders in Tsarang also have higher blood flow as a high-altitude adaptation, similar to other Tibetans. In addition, the mean (95% CI) Hb of Tibetans in the present study was 13.6 g/dl (13.1–14.0 g/dl), which is clearly lower than that of Andeans and almost equal to that of Japanese lowlanders measured using the same device [34]. This finding might indicate that Tibetans living in Tsarang have a low-Hb adaptation to high altitude; however, as some of the present individuals had higher Hb (≥17.0 g/dl) (Fig. 2), it is necessary to assess the mechanism for individual differences in more detail. In the total regression analysis, 22.3% of the variation in SpO_2_ was explained in men and 36.5% in women, but the remaining variation was unclear. Recent studies have reported that the physiological status of highlanders is affected by *EPAS1* and *EGLN1* genes [7, 8, 11, 18, 35–37]. The effect of these genetic variations and other underlying factors on individual variations requires further investigation.

The present study has several limitations. First, the results do not necessarily show a causal relationship because of the cross-sectional design of the study. Second, the sample size was limited and also information on other determinants (e.g., ventilation, nutritional status, or menstrual cycle) contributing to SpO_2_ was not obtained. Third, because Hb concentrations were estimated values, they are difficult to compare with values reported in other studies.

## Conclusion

Among Tibetan highlanders of Tsarang village, higher SpO_2_ showed a weak correlation with lower age and higher Hb in men, and higher SpO_2_ was related to lower heart rate and lower Hb in women. Those living in Tsarang have a low-Hb adaptation to high altitude similar to that of other Tibetans, but Hb was higher in some individuals. These results suggest the presence of individual variations and sex differences in the hemodynamics of high-altitude adaptation in this population.

## Data Availability

Not applicable.

## Abbreviations

95% CI: 95% confidence interval
BMI: Body mass index
DBP: Diastolic blood pressure
EGLN1: Egl-9 family hypoxia-inducible factor 1
EPAS1: Endothelial PAS domain-containing protein 1
Hb: Hemoglobin
SBP: Systolic blood pressure
SpO_2_: Percutaneous arterial oxygen saturation
TAR: Tibet autonomous region
